# Big cohort studies offer insights into preventable risk factors

**DOI:** 10.1093/eurheartj/ehab566

**Published:** 2021-09-04

**Authors:** Karolina Agnieszka Wartolowska, Alastair John Stewart Webb

**Affiliations:** Wolfson Centre for Prevention of Stroke and Dementia, Nuffield Department of Clinical Neurosciences, University of Oxford, Wolfson Building, John Radcliffe Hospital, Oxford OX3 9DU, UK; Wolfson Centre for Prevention of Stroke and Dementia, Nuffield Department of Clinical Neurosciences, University of Oxford, Wolfson Building, John Radcliffe Hospital, Oxford OX3 9DU, UK

**This commentary refers to https://doi.org/10.1093/eurheartj/ehaa756; ‘On cerebrotoxicity of antihypertensive therapy and risk factor cosmetics’, by F.H. Messerli *et al.*, https://doi.org/10.1093/eurheartj/ehaa971; ‘Midlife blood pressure is associated with the severity of white matter hyperintensities: analysis of the UK Biobank cohort study’, by K.A. Wartolowska and A.J.S. Webb, https://doi.org/10.1093/eurheartj/ehaa756 and the discussion piece ‘Cerebrotoxicity of antihypertensive therapy in the UK Biobank Cohort Study’, by F.H. Messerli *et al*., https://doi.org/10.1093/eurheartj/ehab567.**

We would like to thank Drs Messerli, Bavushi, Messerli, and Sionti for their interest^1^ in our study^2^ and for the opportunity to clarify any misunderstandings.

Our paper demonstrates that a large observational cohort study can improve our understanding of modifiable risk factors beyond the timescale that is feasible in most clinical trials, and specifically that midlife DBP is associated with long-term cerebrovascular injury. We agree therefore that our study’s principal implication is that it is important to treat DBP according to guidelines, particularly in midlife, and that in the recent literature the role of diastolic blood pressure (DBP) has often been marginalized. Moreover, white matter hyperintensities (WMH) burden and other consequences of long-term exposure to elevated BP may not be reversible, as reflected in *Figure 2* of our paper demonstrating that even if BP is <130/80 mmHg or even <120/70 mmHg, WMH load is still higher than in untreated people with low BP, supporting the importance of effective early control of blood pressure. However, treatment decisions should be based on randomized evidence and we agree that labelling everyone with BP over 120/70 mmHg as hypertensive may not be helpful and can lead to ‘over-medicalization’, with a potential for harm in some patient groups, as demonstrated in the SPRINT trial.^3^ Therefore, we do not advocate treating BP to the observational targets in the data here, but in accordance with guidelines as supported by clinical trials.

As such, the re-interpretation of our study is surprising. Antihypertensives are not cerebrotoxic, and we specifically did not conclude that they are harmful. This is a misreading of observational data confusing the direction of causality. This was a prospective cohort study, not a randomized-controlled trial; therefore, the treated and untreated participants differed in more ways than by their medication status. So, differences between treated and untreated people do not indicate ‘that antihypertensive treatment per se, independent of its effect on BP, was a powerful risk factor for WMHs’ and cannot be interpreted as ‘antihypertensive therapy must be cerebrotoxic’. It is far more likely that people with established, long-standing hypertension are prescribed antihypertensive medication and the same people have extensive WMH changes.

Our study demonstrated that people who did not require antihypertensive medication, as well as treated people with well-controlled blood pressure, had lower WMH load than those who remained hypertensive despite the treatment. However, the effects of the antihypertensive medication were not the objective of this study but were included in the supplementary data for completeness and openness. As detailed information about specific drugs, their dosage, and dosage changes was not available at the time of this study, we could not specifically investigate the effect of medication. However, as the Editors pointed out, and as has been demonstrated by the SPRINT trial^3^ and a recent systematic review and meta-analysis,^4^ antihypertensive medication strongly modifies the negative cardiovascular consequences of high blood pressure.

The future follow-up data from the UK Biobank cohort may show whether the people with mildly elevated BP, particularly high DBP, in their 40s are more likely to develop vascular dementia.

## Funding

This work was supported by an Alzheimer’s Society grant (450-AS-PG-18-018). AJSW is also funded by a Wellcome Trust Clinical Research Career Development Fellowship (206589/Z/17/Z). The funders of the study had no role in study design, data collection, data analysis, data interpretation, or writing of the manuscript. The corresponding author had full access to all the data in the study and had final responsibility for the decision to submit for publication.

**Conflict of interest:** None declared.

## Data availability

This study used UK Biobank data which are available to eligible researchers after registration.
